# Designing Multi-Antigen Vaccines Against *Acinetobacter baumannii* Using Systemic Approaches

**DOI:** 10.3389/fimmu.2021.666742

**Published:** 2021-04-16

**Authors:** Michael J. McConnell, Antonio J. Martín-Galiano

**Affiliations:** Intrahospital Infections Laboratory, National Centre for Microbiology, Instituto de Salud Carlos III (ISCIII), Majadahonda, Spain

**Keywords:** antibiotic resistance, epitope, multidrug resistance, reverse vaccinology, systems biology

## Abstract

Vaccines and monoclonal antibodies are promising approaches for preventing and treating infections caused by multidrug resistant *Acinetobacter baumannii*. However, only partial protection has been achieved with many previously tested protein antigens, which suggests that vaccines incorporating multiple antigens may be necessary in order to obtain high levels of protection. Several aspects that use the wealth of omic data available for *A. baumannii* have not been fully exploited for antigen identification. In this study, the use of fractionated proteomic and computational data from ~4,200 genomes increased the number of proteins potentially accessible to the humoral response to 8,824 non-redundant proteins in the *A. baumannii* panproteome. Among them, 59% carried predicted B-cell epitopes and T-cell epitopes recognized by two or more alleles of the HLA class II DP supertype. Potential cross-reactivity with human proteins was detected for 8.9% of antigens at the protein level and 2.7% at the B-cell epitope level. Individual antigens were associated with different infection types by genomic, transcriptomic or functional analyses. High intra-clonal genome density permitted the identification of international clone II as a “vaccitype”, in which 20% of identified antigens were specific to this clone. Network-based centrality measurements were used to identify multiple immunologic nodes. Data were formatted, unified and stored in a data warehouse database, which was subsequently used to identify synergistic antigen combinations for different vaccination strategies. This study supports the idea that integration of multi-omic data and fundamental knowledge of the pathobiology of drug-resistant bacteria can facilitate the development of effective multi-antigen vaccines against these challenging infections.

## Introduction

Over the last 40 years *Acinetobacter baumannii* has emerged as a difficult-to-treat pathogen due to the global dissemination of multidrug resistant strains. Bloodstream, lung, urinary tract and wound infections caused by *A. baumannii* occur primarily in the hospital environment, principally in critically-ill patients in intensive care settings (1). However, *A. baumannii* can affect other populations, as antibiotic resistant *A. baumannii* has also emerged as an important cause of neonatal sepsis in developing countries (2–4). Antimicrobial resistance in *A. baumannii* is facilitated by its capacity to form biofilms (5) and genomic plasticity that allows it to acquire resistance determinants (6), including through horizontal gene transfer (7). The treatment of infections caused by isolates with resistance to multiple antibiotic classes, including carbapenems, has been limited to antibiotics that remain active against these strains, such as colistin. Importantly, infections caused by pandrug resistant *A. baumannii*, with resistance to all clinically-used antibiotics have been reported over the last 15 years (8). Limited therapeutic options and associated healthcare costs for these infections have led the World Health Organization to designate carbapenem-resistant *A. baumannii* as having the highest priority (Priority 1: Critical) for the development of novel therapeutics (9).

*A. baumannii* therefore epitomizes microorganisms that require alternatives to small molecule-based antimicrobial therapy (10). Multiple studies have reported protective immunity against *A. baumannii* after prophylactic vaccination or administration of antibodies in rodent infection models (reviewed in (11)). These preclinical results suggest that prophylatic vaccination and passive therapeutics could contribute to the prevention and treatment of infections caused by this pathogen. A key step in the development of vaccines and therapeutic monoclonal antibodies (mAbs) against bacterial pathogens is the identification of antigens that may have increased probability of inducing universal immunoprotection. A monoclonal antibody against the *A. baumannii* capsular polysaccharide was able to rescue infected mice in a lethal sepsis model (12). Candidates that have been tested in preclinical vaccine models include the OmpA porin (13), Ata (14) and Bap (15), and more recently BamA (16) and Omp22 (17) among others (11). However, many of these candidates provide only partial protection against infection, as exemplified by vaccination with the OmpA porin (13). This underscores the intrinsic difficulty in achieving prophylaxis against *A. baumannii*, and warrants the application of more complex and comprehensive antigen identification approaches.

The use of computational methods together with the availability of multiple sequenced genomes for bacterial pathogens has facilitated the rational identification of antigens over reductionist and empirical approaches for vaccine/mAb design, a protocol known as reverse vaccinology (RV) (18). Multiple studies employing different RV methodologies for *A. baumannii* antigen identification have been published since 2013 (19–28). In general, most RV pipelines are based on a reduced number of calculable features subjected to decision schemas or machine learning algorithms (29). However, antigen identification using standard RV pipelines can be limited at several points including: (a) epitopes in bacterial antigens may be present in non-homologous human proteins; (b) promising candidate antigens could be excluded when strict universal presence in the species is used for selection, even when they are absent only in isolates with low clinical interest; (c) pre-existing epitope switch leading to vaccine evasion may be overlooked if sequence identity is only considered at the whole protein level; (d) the expression of many secreted and surface proteins is limited to certain stages of infection (30, 31); and (e) the antigen may exert functions that can be down-regulated or completely halted so that they can be partially substituted by other proteins, thus facilitating vaccine evasion.

The quantity and multiplicity of the omic information available for epitope selection can overcome some of the previously described antigen selection hurdles by extending the RV paradigm to include new criteria, globally termed RV 2.0 (32). The availability of thousands of genomes permits the study of deep antigen occurrence in a lineage-specific manner for international clones of interest. Syndrome-oriented epidemiological trends can be tracked by using Multilocus sequence typing (MLST) schemas (33, 34), the Biosample database (35), and the characterization of antigen expression programs can be determined by transcriptomic studies (36). Computational studies that identify antigens for vaccine development often produce ranked lists of candidates for monovalent vaccine development. However, the fact that highly antigenic proteins can be subjected to evolutionary forces that select for their mutation, down-regulation or elimination may hamper the long-term effectiveness of vaccines based on a single protein antigen. These individual antigens can be combined experimentally and tested to identify highly effective antigen combinations. Multi-omic analyses employing systems biology approaches may facilitate the selection of optimized antigen cocktails that include proteins, which can induce a synergistic immune response.

In the present work, we build upon previous studies that have identified antigens in *A. baumannii* by employing RV 2.0 concepts in the analysis of genome sequence data from more than 4,100 *A. baumannii* isolates. We have incorporated multiple variant-associated metadata and experimental datasets from whole organism profiling studies in order to incorporate clonality and expression under disease-like conditions in an antigen identification pipeline. Finally, we employ this pipeline to identify antigen combinations for the development of multi-antigen vaccines that respond to two different vaccination strategies, namely “siege” and “exhaustion” strategies.

## Materials and Methods

### Proteome Acquisition and Management

*A. baumannii* proteomes corresponding to RefSeq complete and draft genomes were downloaded from the Assembly NCBI database (Status: February 21st 2020) (37). Proteins sequences were clustered using CD-HIT applying 90% identity and 90% alignment coverage (37). Protein cluster representatives were assigned to clonal STs, if available, in >80% of the isolates of such clones and the total is more than 5 isolates. The MLST sequence type (ST) clone for an isolate was identified by BLAST by applying the thresholds of 100% identity, 100% alignment coverage to allelic sequences of the Oxford schema from the MLST database (33). International clones were those deemed by the Pasteur MLST schema, utilizing ST81 for IC-1, ST2 for IC-2 and ST3 for IC-3, plus their respective single locus variants.

### Detection of Exposed Proteins

Proteins exposed on the bacterial surface were detected by three strategies. First, the presence of signal peptide of proteins exported to the cell wall or the outer space was predicted using SignalP 5.0 and applying the “Gram-negative” training and default parameters (38). Second, surface proteins were also identified through detection of Pfam domains associated with surface complexes using *hmmscan* of the HMMER 3.0 suite applying gathering thresholds (39). Finally, proteins detected in the exposed sub-cellular fractions of proteomic datasets were also considered as exposed proteins. Equivalent surface proteins to those reported in proteomic experiments were identified in the non-redundant dataset by the BLAST hit with the highest score and E-value < 10^-5^.

Soluble expression of recombinant proteins in *E. coli* was predicted by Soluprot 1.0 (40). Transmembrane helices were predicted with TMHMM 2.0 (41). Allergenicity was predicted by AllerTop 2.0 (42) and the five methods available in AlgPred 2.0 (43). Toxicity was predicted with ToxinPred (44) using the “SVM (Swiss-Prot) + Motif based” method.

### Epitope Prediction

Linear B-cell epitopes were predicted by the consensus of five tools implemented by the Immune Epitope Database (45), which include beta-turn prediction (46), surface accessibility prediction (46), flexibility prediction, antigenicity (47) and hydrophilicity prediction (48).

Peptide ligands to human leukocyte antigen (HLA) class II molecules were predicted by NetMHCIIpan 3.2 (49) and the consensus of three other methods (50). The 15mer high-affinity epitopes showing ic50≤100nM and/or in the top-2 adjusted rank percentile were selected and further merged if overlap of at least one residue into longer epitope spans. The alleles of HLA class II supertype consisting of DPB1*0101, DPB1*0201, DPB1*0401, DPB1*0402 and DPB1*0501 alleles (51), were selected.

Perfect epitope conservation was checked in proteomes of 118 *Acinetobacter nosocomialis* and 48 *Acinetobacter pittii* isolates, available in the Assembly NCBI database, by an in-house perl script.

### Antigen Expression Associated With Infection Type

Isolates were associated with seven specific infection types using Biosample records through the “isolate source” and then “host disease” metadata fields by a manually curated dictionary that unifies 202 distinct medical terms, synonyms and typos. Only one representative isolate per ST (Oxford schema) and syndrome was considered. The relative frequency of the 8,824 non-redundant exposed proteins in each syndrome-linked isolates was calculated. Proteins in none or all syndromes (i.e. in ≥95 of isolates of the syndrome) were rejected, which yielded 311 “inter-syndrome variable” proteins. Bidimensional arrays with the seven syndromes and the relative frequency for the 311 proteins were built. Hierarchical clustering of syndromes according to this protein dataset was carried out using R environment. Data was normalized with the scale function, distances were calculated with the “euclidean” method of the dist function, and the hierarchical clustering carried out with the “ward.D2” method of the hclust function.

### Virulence and Interactomic Analyses

Virulence factors in our non-redundant exposed protein dataset were detected by BLAST to the Virulence Factor Database (VFDB) (52) and Victors (53) database contents applying an identity threshold of ≥50% over bidirectional ≥60% alignment length. A threshold of 60% mutual alignment coverage was employed as it allows for the selection of the domain architecture core, as the function of multi-domain proteins (as many virulence proteins are) ultimately relies on the synergistic sub-functions exerted by the constituting domains. Globally, the double threshold applied for virulence protein detection satisfied the assumption that hits likely maintain the virulence-associated functions of the homologs stored in the virulence databases. Interactomic-based essentiality analyses was carried out using protein-protein link data provided by the STRING DB v11 (54) and applying the confident value of ≥0.7 (“highly confident” according to the STRING DB v11 threshold classification) for the combined STRING score. STRING sequences were assigned to non-redundant exposed proteins by BLAST using ≥80% identity and sequence coverage of ≥60%. Degree centrality and median betweenness centrality were calculated using the *degree_centrality* and *betweeness_centrality* methods of the Networkx python library, respectively. In particular, betweenness centrality was calculated as the fraction of shortest paths that pass through each node.

## Results and Discussion

### Global and Comparative View of Previous Reverse Vaccinology Approaches for *A. baumannii*

**Figure 1:**
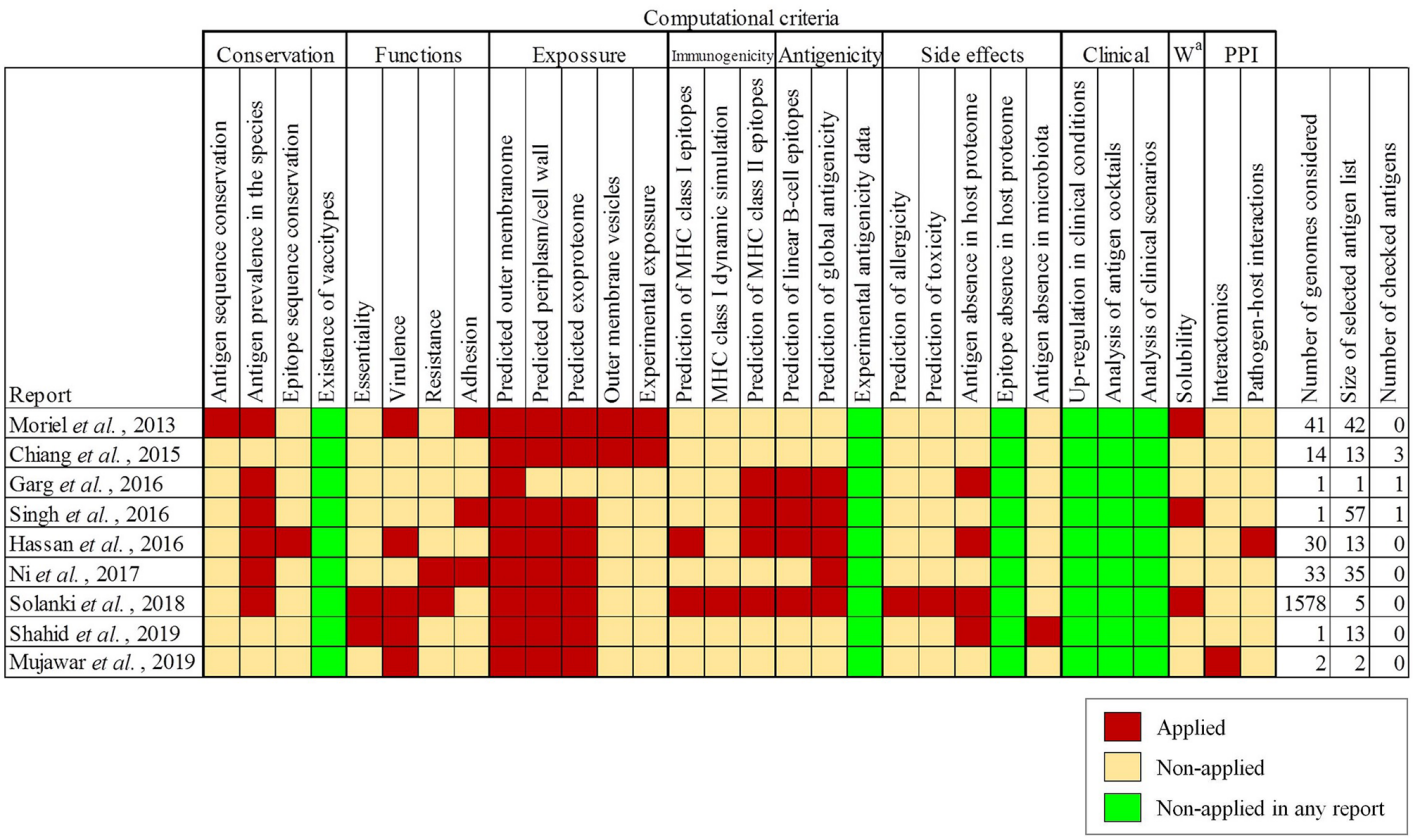
Antigen selection criteria applied in previously published studies employing RV techniques for antigen identification for *A. baumannii*. Criteria for antigen selection are indicated and grouped by topic. ^a^ Workability.

To determine to what extent the current literature covers different aspects employed in antigen selection in *A. baumannii*, a meta-analysis of previous studies employing computation approaches was carried out. This identified ten reports that together covered 24 different computational criteria used for antigen identification and prioritization (19–28). Most of these studies employed less than 100 genome sequences for which intra-species prevalence, protein location and cellular/humoral antigenicity were evaluated (**Figure 1**). Prediction of toxicity, allergenicity and potential host self-immunity, are also considered by some approaches. Two recent studies (25, 28) identified highly conserved epitopes present in outer membrane antigens for development of chimeric multi-epitope vaccines. One study further focuses on protein network position and participation in different physiological processes (27). Two studies reported the use of the Vaxign platform for antigen selection (55). The different selection criteria and assumptions applied in these studies to a limited number of genome sequences yielded lists with different numbers (1-57) of high-quality antigens as protein vaccine candidates, from which a small number (0-3) were experimentally validated. Importantly, in our view, six conceptual questions remain to be explored in depth (**Figure 1**, green columns). We have approached these aspects in the following sections.

### Maximal-Inclusivity of the Surface Exposed *A. baumannii* Proteome

**Figure 2:**
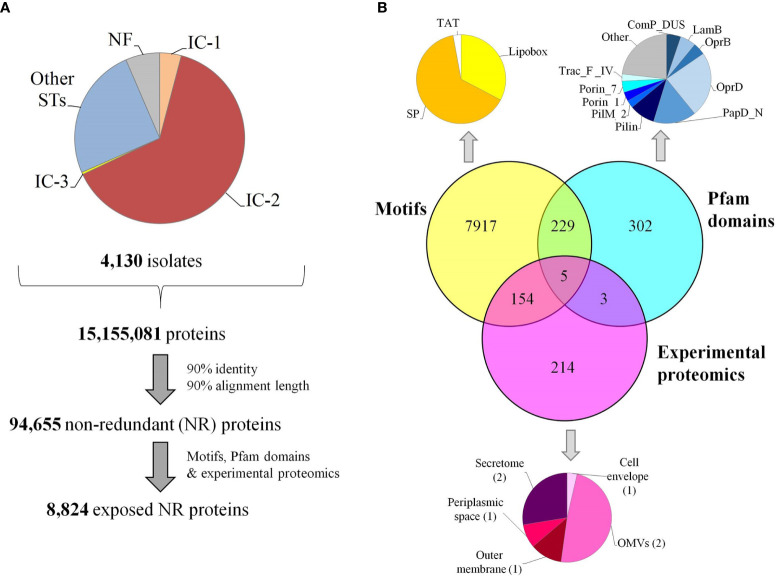
Pan-proteome data management and selection of exposed proteins. **(A)** Flowchart indicating pan-proteome data collection, redundancy reduction and exposed protein selection. Genomic representation of international clones is shown. **(B)** Identification of exposed proteins by three approaches. OMVs, outer membrane vesicles; SP, signal peptides transported by the Sec translocon; TAT, signal peptides transported by the Tat translocon.

Bacterial antigens must be accessible to immune system effectors. To achieve maximal inclusivity of surface exposed antigens, two strategies were employed. First, all Assembly database content for *A. baumannii* consisting of 4,170 isolates was considered. This analysis included 2.7-fold more isolate information than the largest genome dataset previously published for *A. baumannii* RV studies (25). Up to 84 ST (Oxford schema) with ≥5 isolates were represented of which 4.1% belonged to International Clone (IC) I, 63.9% to IC-2 and 0.4% to IC-3 (**Figure 2A**). The pan-proteome dataset included ~15.1 million proteins, representing the known protein universe of this species. This was reduced to 94,655 representative proteins based on a 90% identity and 90% alignment coverage thresholds. We considered these parameters reach a reasonable balance between ortholog relatedness and immunological redundancy within protein families in the pan-proteome.

Second, three independent factors providing evidence of exposition in the non-redundant dataset were taken into account: (a) the detection of universal motifs (i.e. SP/TAT signal peptides and lipobox), (b) the presence of any of 50 pfam motifs associated with known surface complexes (i.e. flagella, pili and porins) (**Supplementary Table S1**), and (c) proteins detected in exposed subcellular fractions revealed by seven experimental proteomic assays (**Supplementary Table S2**) (**Figure 2B**). As a result, 8,824 representative proteins (9.3% of the dataset) were deemed exposed, which corresponds to approximately 400-500 proteins per isolate.

### Antigenicity of the Exposed Proteome

**Figure 3:**
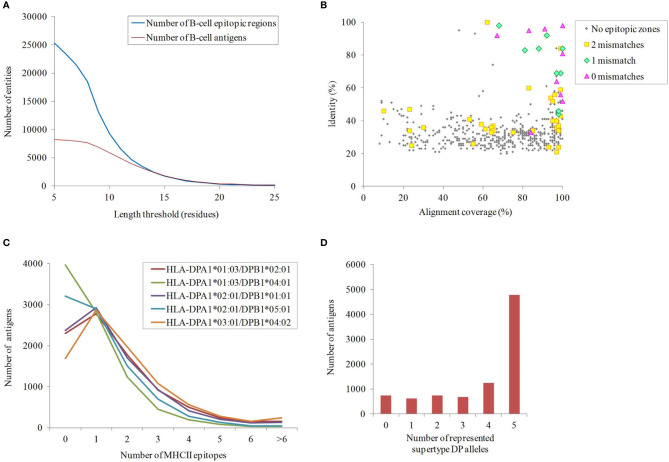
Immunogenicity of the exposed *A. baumannii* proteome. **(A)** B-cell epitope spans with different lengths predicted by ≥4 methods. **(B)** Antigens potentially shared with the human proteome. The identity and alignment coverage of the best BLAST hit to human proteome ranked by score. Those *A. baumannii* proteins with human homologs and epitope zone matching with ≤ 2 mismatches are indicated. **(C)** Number of predicted binding HLA class II epitopes for the five DP supertype alleles per exposed proteins. **(D)** Cumulative number of exposed proteins according to the carried HLA class II alleles of the DP supertype.

Although protein exposure on the bacterial surface is a requirement, it is not sufficient for ascertaining antigenicity. Prediction of B-cell epitopes, especially with well-defined limits, is extremely challenging (56). Nevertheless, exposed regions showing particular physicochemical properties are indicative of antibody recognition. Sections of ≥10 residues fulfilling at least four out of the following criteria: beta-turns, flexibility, hydrophilicity, surface accessibility and general antigenicity, were found in 66.7% proteins in our dataset (**Figure 3A**). Remarkably, 32.5% and 28.9% of these B-cell epitopic zones were perfectly conserved in some *A. nosocomialis* and *A. pittii* isolates, respectively, two opportunistic hospital pathogens related to *A. baumannii*.

The potential for undesired cross-reactivity between antibodies against antigens in our dataset and human proteins was investigated. A total of 555 non-redundant exposed A*. baumannii* proteins carrying B-cell epitope zones showed significant similarity (E-value < 10^-5^) to proteins in the human proteome. This was further scrutinized at the epitope level. Nine antigens carried predicted B-cell epitope zones identical to sequences in human proteins, whereas 14 antigens contained epitope zones with one mismatch compared to human sequences, and 235 with two mismatches. All epitopes showing a perfect match were carried by antigens with human homologs but, strikingly, only 64% and 14% of *A. baumannii* antigens with one or two mismatching epitopes, respectively, corresponded to full-length human homologs (**Figure 3B**). These data underscore the importance of assessing human cross-reactivity at both whole protein and epitope levels in RV pipelines.

Exposed proteins with the ability to universally stimulate T-helper lymphocytes *via* HLA class II may be more effective antigens. T-helper lymphocytes orchestrate a mature response through numerous effector functions that clears bacterial infections (57). Given that antibodies against outer membrane antigens has been shown to be sufficient for protection against *A. baumannii* in animal models (14, 58, 59), in this study we focused on the identification of epitopes presented through HLA class II pathway. Epitopes presented through this pathway are typically 15 amino acids in length, so this peptide size was used in this study. The importance of the cytotoxic response in a non-intracellular as *A. baumannii* is likely marginal and thus HLA class I epitopes were not considered. In order to identify antigens that activate this pathway, the presence of epitopes predicted to bind the five HLA class II alleles of the DP supertype, which cover ~90% human population regardless of ethnicity (51), was computationally screened (**Figure 3C**). Up to 84.6% of the proteins carried antigens for at least two alleles of the DP supertype (**Figure 3D**). Among these, 5,182 exposed proteins also contained at least one B-cell epitope. These included homologs for 11 out of the 13 proteins previously identified as antigens with the ANTIGENome technique (60). Exact matches for 24.4% and 24.9% of these HLA class II epitopes were also found in some *A. nosocomialis* and *A. pittii* isolates, respectively. Overall, data suggest cross-protection between *A. baumannii* and related species may be obtained.

### Identification of Antigens Associated With Specific *A. baumannii* Infection Types

Individual antigens may be associated with different infection types. This raises the possibility that vaccine antigens could be targeted to specific syndromes among those caused by *A. baumannii*. Conversely, the identification of antigens involved in multiple types of infections may facilitate the development of broadly active vaccines.

**Figure 4:**
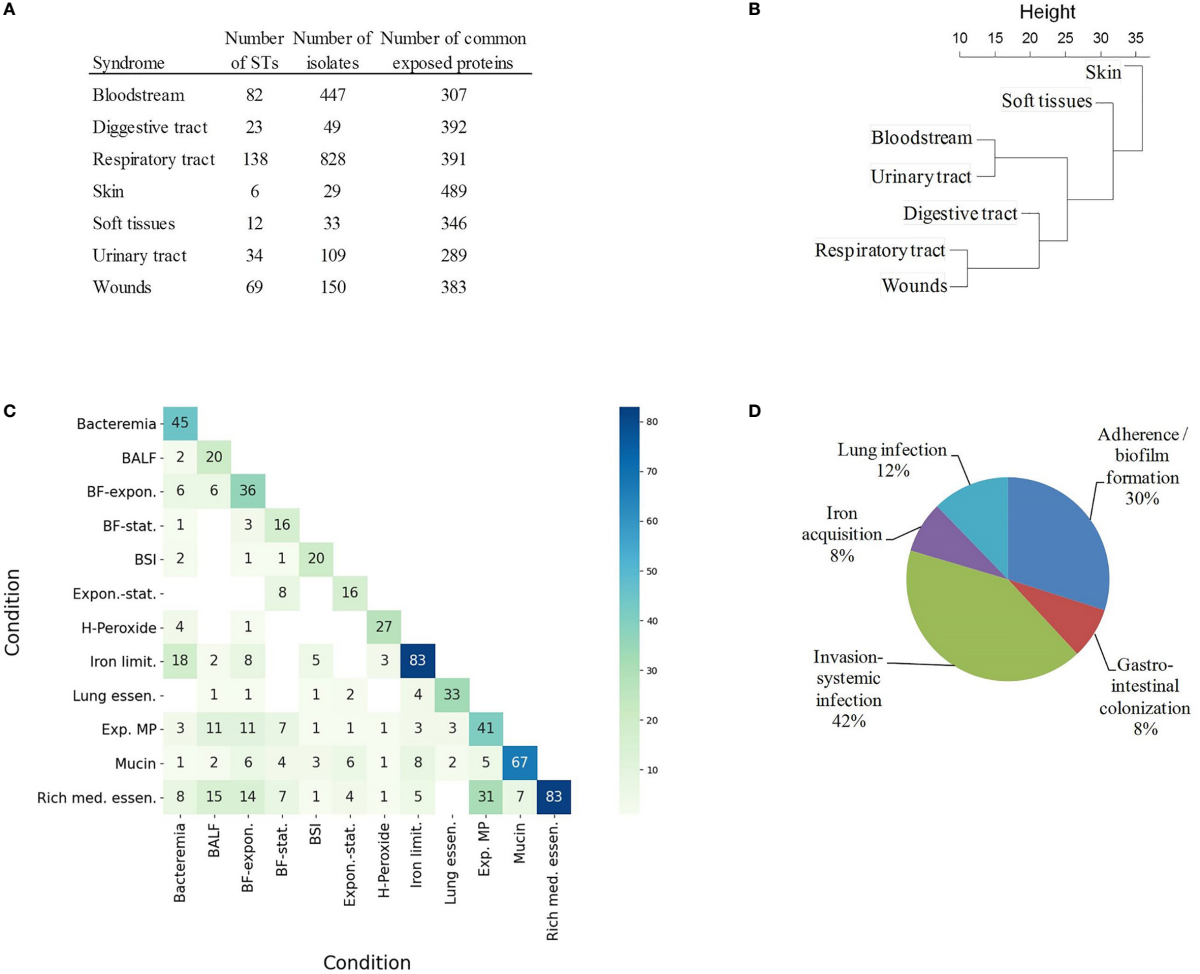
Coincidental degree of gene upregulation between conditions. **(A)** Table of STs and number of isolates associated to syndromes in Biosample and their common exposed proteomes. **(B)** Hierarchical tree using the relative occurrence of exposed proteins with occurrence variability between syndromes. **(C)** Heatmap indicating the absolute number of matching exposed proteins between conditions. BALF, Exposure to bronchoalveolar lavage fluid; BF-expon., Biofilm vs exponential planktonic cells; BF-stat., Biofilm vs stationary planktonic cells; BSI, Bloodstream infection; Expon.-stat., Exponential vs stationary planktonic cells; H-Peroxide, Hydrogen peroxide 5mM; Iron limit., Iron limitation; Lung essen., Lung essentiality; Exp. MP, Exposure to macrophages; Mucin, 0.5% mucin in swimming broth; Rich med. essen., Rich medium essentiality. **(D)** Relative proportion of exposed proteins according to functional class in VFDB and Victors databases.

In order to explore this aspect, the antigenic content of 1,656 isolates (39.7% of the dataset) sampled from infected sites was compared. Isolates were assigned to seven infection types using Biosample records (see Materials and Methods) (**Figure 4A**). Bias due to clone prevalence in the dataset was minimized by selecting only one representative isolate per ST (and one non-typeable) and per syndrome. Each infection type was linked to 289-489 exposed proteins present in ≥ 95% of isolates. Among these proteins, 311 did not reach the ≥ 95 isolate occurrence in at least one syndrome. The normalized occurrence data of these antigens was used to build a hierarchical tree that depicts the proteomic relationship between different infection types (**Figure 4B**). Clades were observed for bloodstream and urinary tract infections on the one hand, and respiratory tract and wound infections on the other hand, whereas skin colonization was far from both clades. However, only fifteen proteins reached statistical significance by chi-square test (**Supplementary Table S3**); for association with a particular infection type. All identified antigens exerted unknown functions except for the lysozyme inhibitor LprI (formerly DUF1311), which may confer some advantage in the macrophage environment (61), the iron acquisition protein Ton-B; and YgiW/YdeI, which protects against antimicrobial peptide stress (62). This weak proteome-infection type association is, very likely, a consequence of the fact that a majority of isolates can cause different syndromes. This may result from genome-independent factors such as patient, environmental and seasonal variables.

Antigens specific to different infection types can also be identified experimentally in studies that characterize conditional antigen expression in different conditions. To explore this aspect, our non-redundant exposed antigen dataset was compared to essential and upregulated genes identified in published studies during bacterial growth in 12 conditions that mimic different infection types (**Supplementary Table S4**). The intersection of exposed proteins between “rich-medium essentiality” and “exposure to macrophages” (Jaccard index (J) = 0.50), “exposure to bronchoalveolar lavage fluid” (J = 0.29) and “sessile-to-planktonic states” (J = 0.24) was notable (**Figure 4C**). Bacteremia and “iron limitation” were also very coincidental (J = 0.28). On the other hand, mucin exposition was an outlying condition in our analysis (J ≤ 0.12 with respect to the rest of conditions). Mucin is a rich source of amino acids and metals (63), in contrast to bacteremia, during which free iron is heavily restricted.

Finally, syndrome-associated data was complemented by integration into a pathofunctional context. A total of 150 exposed proteins had homologs in the VFDB and Victors databases, which store virulence factors that play critical roles during infection or were identified as proteins that participate in virulence experimentally. A number of adhesion, capsule expression, iron acquisition and metabolic factors were identified (**Figure 4D**).

### Antigen Presence at the Intra-Clonal Level

**Figure 5:**
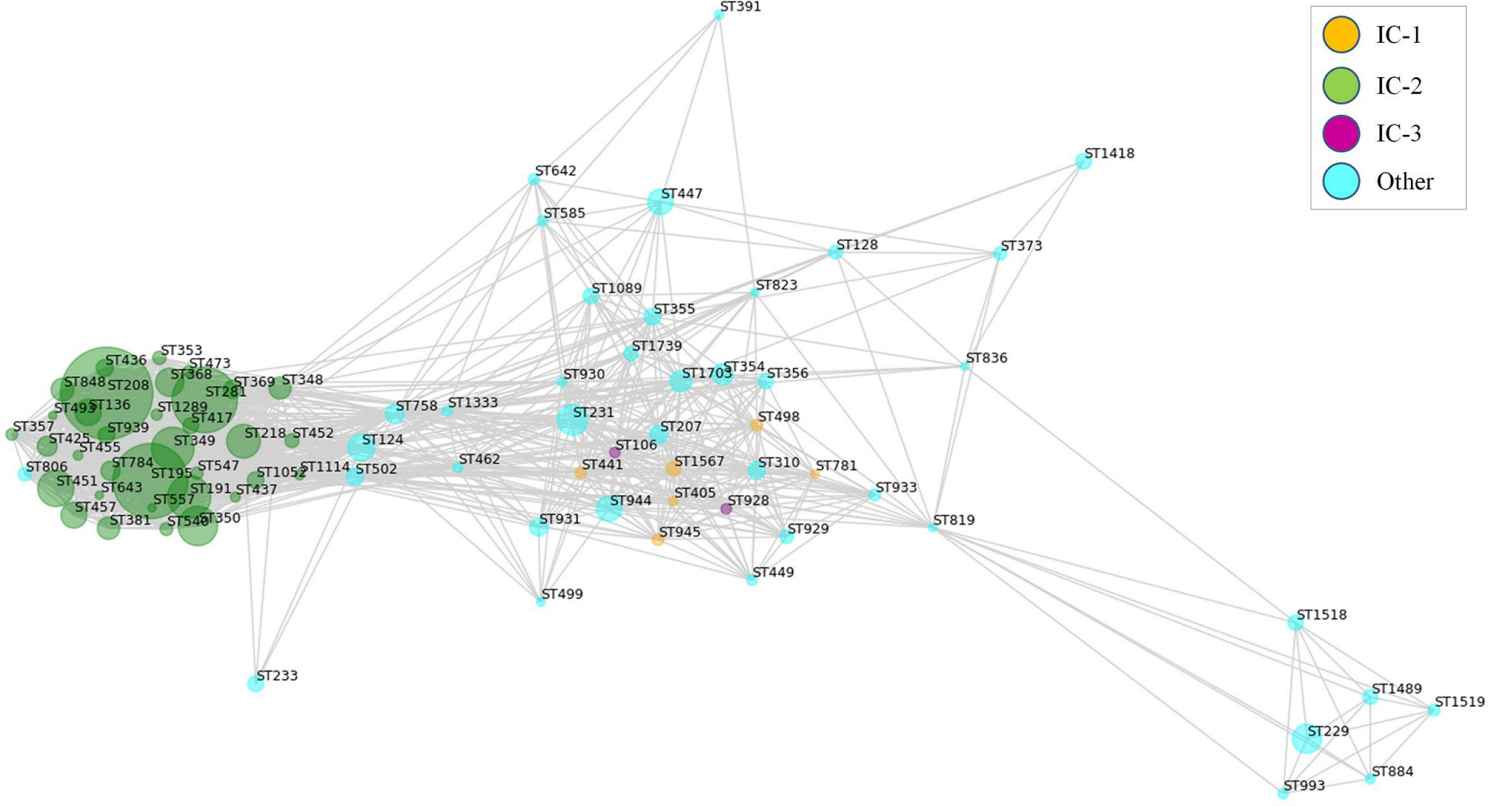
Weighted map showing clonal-dependence of the predicted epitope content in the exposed proteome. Exposed proteins were considered represented in STs with ≥5 isolates if ≥80% isolates in the ST harbored the protein. Proteins present in all STs were not considered. Predicted B-cell epitopic zones (≥4 methods and ≥10 residues) for the ST-associated proteins were extracted (805 ± 35 epitopes [average ± SD] per ST). All STs were subsequently compared by pairs according to their B-cell epitope-zone pools. Jaccard distances were calculated considering the number of non-shared epitope zones with respect to the total in the ST pair. Only ST pairs with Jaccard distances of ≤0.25 were considered for graph elaboration. Sphere diameter is proportional to the number of ST isolates (ranging from 5 to 622). Sphere color represents the international clone (see Legend). The network was constructed using the python *Networkx* library.

RV approaches have often used universal antigen distribution over the whole species as a fundamental criterion for antigen selection. However, for many bacterial species the majority of clinically relevant infections are often caused by only a few clones. Thus, it may be reasonable to focus antigen identification approaches on these clinically relevant clones. In the Assembly database, 84 A*. baumannii* STs were represented by ≥5 sequenced isolates, which can be employed in the analysis of the exposed proteome of the species at an intra-ST level. Map depiction using distances between antigenic content in these groups yielded a central subnetwork populated by many STs including those from IC-1 and IC-3 (**Figure 5**), in addition to multiple satellites. Notably, STs pertaining to IC-2, the *A. baumannii* lineage accounting for most sequenced isolates and multidrug-resistance cases (64), grouped into its own subnetwork. These data strongly suggest the existence of *A. baumannii* lineages that experienced a partial immunological independence, i.e. can be deemed “vaccitypes”, from which IC-2 clearly presents itself as an attractive target.

### Position of the Antigen in the Exposed Protein Network

**Figure 6:**
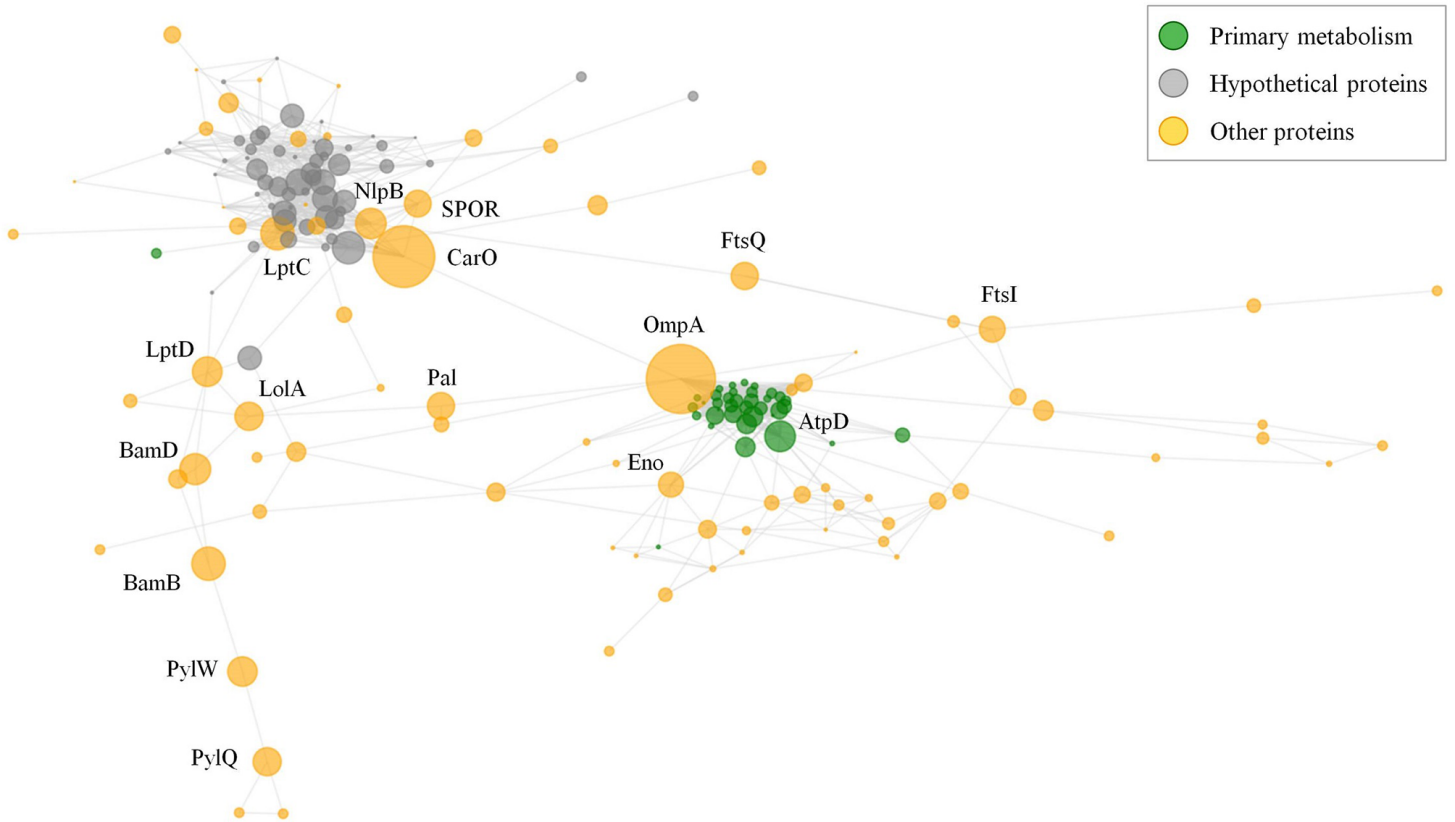
Exposed interactome of *A. baumannii*. Non-weighted undirected graph of the exposed proteome is shown. Only proteins with betweenneess-centrality >10^-5^ were selected (174 proteins). Sphere diameter is proportional to betweenneess-centrality. Node color indicates protein functional class (see legend). Protein labels are provided for proteins with betweenneess-centrality ≥ 0.02. The graph was depicted with python *matplotlib* library methods.

A common challenge for the development of vaccines and mAbs is the rational detection of antigens whose inactivation (in isolation or in group) cannot be easily tolerated by the targeted pathogen. Although two previous studies have evaluated network data in *A. baumannii* (27, 28), we aimed to explore the cooperative nature of antigens that could be employed in multi-antigen vaccines using our data set. Selection of effective antigens was therefore evaluated through non-directed graph modeling of the exposed interactome. The network consisted of two evident dense sub-networks plus numerous additional nodes (**Figure 6**). The relevance of each independent node in the structural integrity of this network was evaluated by calculating the metrics of degree- (fraction of directly connected nodes) and betweenness- (number of shortest paths between all node pairs passing across a particular node) centralities. Degree-centrality was high for primary metabolism nodes such as elongation factors and ribosomal proteins, which may be present on the surface participating in “moonlight activities”. However, proteins showing high values for betweenness-centrality are expected to hinder cellular physiology as a whole when they are targeted by antibodies by disjointing the cooperativity between protein subnetworks. Among these, OmpA showed very high betweenness-centrality, as previously reported (27). Nevertheless, there are other cases also related to genuine virulence: the outer membrane assembly protein Bam, important in pellicle formation (65); the abundant CarO porin involved in the transporter of ornithine (66); and the lipopolysaccharide ABC exporter Lpt. Strikingly, some antigenic hypothetical proteins also played moderate central roles (**Figure 6**, grey color).

### Pipeline Construction and Testing

The omic data and bioinformatic predictions described above were formatted, integrated and stored in a MySQL data warehouse database. Based on this, a computational pipeline was built that accepts different dynamic selection settings that can be used to interrogate several antigen selection schemes. The pipeline was then employed to identify antigens that could be applied to different vaccination scenarios for *A. baumannii*.

First, the number of antigens present in the majority (≥ 90%) of the isolates in the species and with no predicted cross reactivity with the human proteome, was evaluated at different thresholds. Up to 249 exposed proteins carried one B-cell epitope zone and covered one HLA class II DP supertype allele, but only 68 and 12 proteins satisfied these thresholds when established in 3 and 5 epitopes/alleles, respectively (**Supplementary Figure S1A**). The later conditions maximize population coverage in terms of host and pathogen species. The number of included antigens sharply dropped when centrality thresholds were applied even at the lowest immunogenicity level. In fact, only five antigenic proteins showed ≥ 10 interacting partners and betweenness-centrality ≥ 0.02 (**Supplementary Figure S1B**, **Supplementary Table S5**).

Given its high clinical importance, antigen independence was assessed only for IC-2. Up to 24% more antigens were found at several epitope stringency levels when the search (presence in ≥ 90% isolates) was restricted to this clone (**Supplementary Figure S1A**). This suggests that if the “isolate occurrence” parameter includes clones that are not clinically relevant, important targets for prevalent lineages may be excluded. Moreover, immune responses against clone-specific antigens may act as “magic bullets” that do not affect the microbiota and avoid potential dysbiosis. In contrast, this clonal effect flattens when more centrality is demanded of antigens (**Supplementary Figure S1B**), indicating that immunohubs are a feature of the species rather than of a single clone. Among the most antigenic and/or central proteins, EscC from the Type III secretion system (T3SS), OmpA and a SPOR domain protein involved in cell division (67) were prioritized.

We next employed our pipeline to identify antigen combinations that could facilitate two different protective procedures, namely the “siege” and the “exhaustion” strategies. During the “siege” strategy, all important antigens required for a known physiological function can be collectively blocked so that the pathogen would not be able to circumvent the immunologic defense. Given current knowledge of the *A. baumannii* infectious process, two pathogenic functions were targeted to explore this hypothesis (**Table 1**): (1) adherence and biofilm formation, a prerequisite to developing further diseases that rendered five proteins by our system; and (2) iron acquisition, an essential function for *A. baumannii* growth and survival *in vivo*. Our pipeline identified five antigens involved in adherence and biofilm formation, and 10 transporters involved in iron acquisition, including several TonB siderophore receptor homologs, two porins and two membrane transporters.

**Table 1:** Antigen list for “iron acquisition” and “adhesion” targets following the “siege” strategy.

Target	Protein	Occurrence (% isolates)	Number of B-cell epitopic zones	Number of DP supertype alleles (epitopes)	Description
Adhesion-biofilm	WP_000777882.1	96.87	5	2 (3)	Membrane protein (OmpA family)
	WP_004644147.1	99.37	1	5 (23)	Pilus assembly protein PilM
	WP_096903805.1	94.96	1	5 (43)	Poly-beta-1,6 N-acetyl-D-glucosamine export porin PgaA
	WP_001061322.1	97.50	3	5 (41)	Poly-beta-1,6-N-acetyl-D-glucosamine N-deacetylase PgaB
	WP_017386534.1	98.74	1	5 (22)	RND transporter
Iron acquisition	WP_000871878.1	99.27	1	5 (45)	Cation acetate symporter
	WP_050675416.1	98.72	1	5 (8)	Ferric anguibactin-binding protein
	WP_000777882.1	96.87	5	2 (3)	Membrane protein (OmpA family)
	WP_000848134.1	99.59	3	4 (5)	Porin (OprB familiy)
	WP_000413985.1	95.54	4	4 (9)	TonB-dependent receptor
	WP_115431403.1	97.17	6	5 (24)	TonB-dependent receptor
	WP_000364460.1	95.32	6	5 (18)	TonB-dependent siderophore receptor
	WP_000831228.1	97.04	7	4 (7)	TonB-dependent siderophore receptor
	WP_001189913.1	95.20	1	5 (13)	TonB-dependent siderophore receptor
	WP_079746199.1	90.94	4	5 (10)	TonB-dependent siderophore receptor

**Figure 7:**
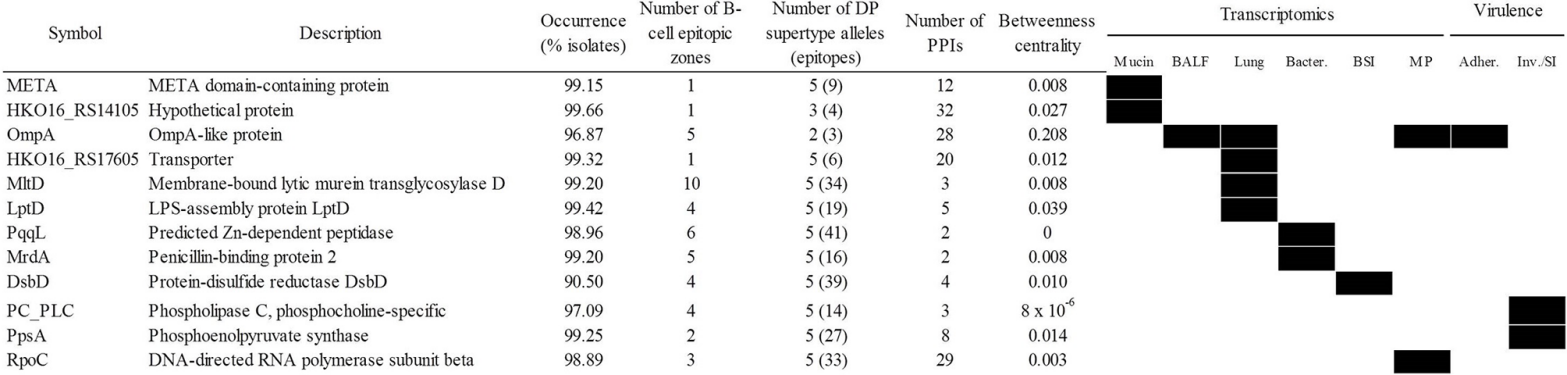
Antigen candidates for “exhausting” vaccination strategies. Upregulation/essentiality of the antigen during infection stages is shown (black rectangles). Adher., adherence; BALF, bronchoalveolar lavage fluid; BSI, bloodstream infection; Inv/SI, invasion/systemic infection; MP, exposure to macrophages. PPIs, protein-protein interactions.

A potential weakness of the “siege” strategy is that if the pathogen is able to overcome the targeted function, it may be able to cause disease without further opposition. Applying the alternative “exhaustion” strategy, the most relevant antigens involved in several functions necessary for infection would be selected, with the aim of acting on the infection pathogen at multiple points during the infectious process. In this respect, the most stringent selection criteria were applied to identify determinants that were universal, central, pluri-epitopic and play important roles in sequential infective steps, comprising respiratory and bloodstream infections. That accounted for 12 proteins (**Figure 7**) including LptD and OmpA. Since the elicited defense using this approach aims to attack several different mechanisms, in our view, the bacteria would have difficulty in bypassing all steps targeted using this strategy. Finally, 24 antigenic proteins were identified as essential in rich medium (**Supplementary Table S6**). However, many of these proteins play conserved housekeeping roles and therefore its utilization as antigens may interfere with the microbiota.

A central ultimate goal of RV is to create subunit vaccines, i.e. using purified recombinant proteins. Therefore, the experimental behavior of the 25 proteins derived from the “siege” and “exhaustion” strategies mentioned above was predicted and studied (**Supplementary Figure S2**, **Supplementary Table S7**). These proteins contained no or very low transmembrane helix content but most showed large sizes, which may affect purification performance. Importantly, four proteins rendered decreased predicted solubility (Soluprot score < 0.3) in *Escherichia coli*. These biochemically recalcitrant polypeptides may anyway be to either re-folding from inclusion bodies or to approximations that involve epitope excision. In addition, potential side effects in host, i.e. allergenicity and toxicity were also analyzed (**Supplementary Table S8**). Only PC_PLC was confirmed as potentially allergenic by both AllerTop 2.0 and AlgPred 2.0 (at least by two AlgPred methods out of five). Twelve proteins carried regions identified as toxic by ToxinPred but such zones only covered < 7.1% of the protein length.

Taken together, the data presented here indicate that several antigens combinations can be identified based on different vaccination strategies. Nevertheless, a standard all-purpose combination that is balanced among several selection schemas would include the best individual antigen (OmpA) reinforced with other selected antigens. The cocktail should include protection during all infection stages, high species coverage and sequence conservation, strong B-/T helper antigenicity and central position in the surface protein network. Using the information of **Figure 7**, we propose that the META-MltD-MrdA-OmpA-RpoC combination would satisfy these goals.

## Conclusions

Widespread *A. baumannii* infections recalcitrant to antimicrobial chemotherapy raised interest in the development of vaccines and mAb therapies. The versatile pathogenicity of this microorganism and previous studies indicate that efficient universal vaccination using a single antigen may be difficult to achieve. Multivalent vaccine formulations or mAbs cocktails tailored to synergistically target critical aspects of infection may facilitate the development of more effective immunity based approaches.

The number of antigens that can be used at once is limited in practice. Here, numerous antigenic cocktails identified using an integrative multi-omic approach are proposed. The wealth of sequenced genomes permitted clonal (STs) and supra-clonal (international clones) considerations to be included. This is appropriate for assessing to what extent lineages that monopolize clinically relevant infections over a given timeframe are immunologically distinct, *i.e.* vaccitypes. Syndrome-oriented combinations can be assessed at genomic, transcriptomic and pathofunctional levels. However, in this respect, the effect of the genomic content that dictates infection type was rather minor, likely due to pathogen-independent factors.

Our study is subjected to limitations intrinsic to the complexity of using systemic approaches in the development of vaccines against bacterial pathogens. Whereas most studies have concentrated on the analysis of antigens in isolation, the body of knowledge regarding rational design of antigen cocktails is scarce. Thus, feedback from dedicated experimental assays are needed in order to refine the combination selection rules. In particular, antigen expression data and detailed molecular studies characterizing the role of potential *A. baumannii* antigens are limited compared to other multi-drug resistance species such as *Staphylococcus aureus* and *E. coli*. Thus, the efficacy of synergic antigen sets would also benefit from increased knowledge of the fundamental physiology of this microorganism. In addition, software grouping all immunoinformatic and genomic tools required for these types of analyses is still in their infancy. Intuitive and highly portable program suites will be developed in future for console and windows-based environments.

Data warehouse databases facilitate the integration of multiple source information to interrogate these complex clinical scenarios. We envisage this will be a hallmark of RV 2.0 approaches in the postgenomic era and the most rapid way to design high-quality cocktails that provide efficient protection. The possibility of evasion by the targeted pathogen may be reduced when combinations of hard-to-substitute antigens identified through the use of several computational approaches are employed. Antigen subsets provided in this study would constitute the starting material for experimental verification in preclinical models.

## Data Availability Statement

The original contributions presented in the study are included in the article/**Supplementary Material**. Further inquiries can be directed to the corresponding authors.

## Author Contributions

MM and AM-G planned the manuscript content, made the formal analyses, wrote the manuscript, and approved the final version. All authors contributed to the article and approved the submitted version.

## Funding

This research was supported by Acción Estratégica en Salud from the ISCIII, grants MPY 380/18 and MPY 509/19. AM-G is the recipient of a Miguel Servet contract by the ISCIII.

## Conflict of Interest

MM is a founder and shareholder in the company Vaxdyn, S.L.

The remaining author declares that the research was conducted in the absence of any commercial or financial relationships that could be construed as a potential conflict of interest.
