# Supplementary material for: Designing Multi-Antigen Vaccines Against Acinetobacter baumannii Using Systemic Approaches
Source: Front Immunol. 2021 Apr 16;12:666742. doi: 10.3389/fimmu.2021.666742 (PMC8085427; doi:10.3389/fimmu.2021.666742)
Supplement: Supplementary file 1 [file Image_1.pdf]

Figure S1. Number of antigens after applying different criterion stringency for selection.

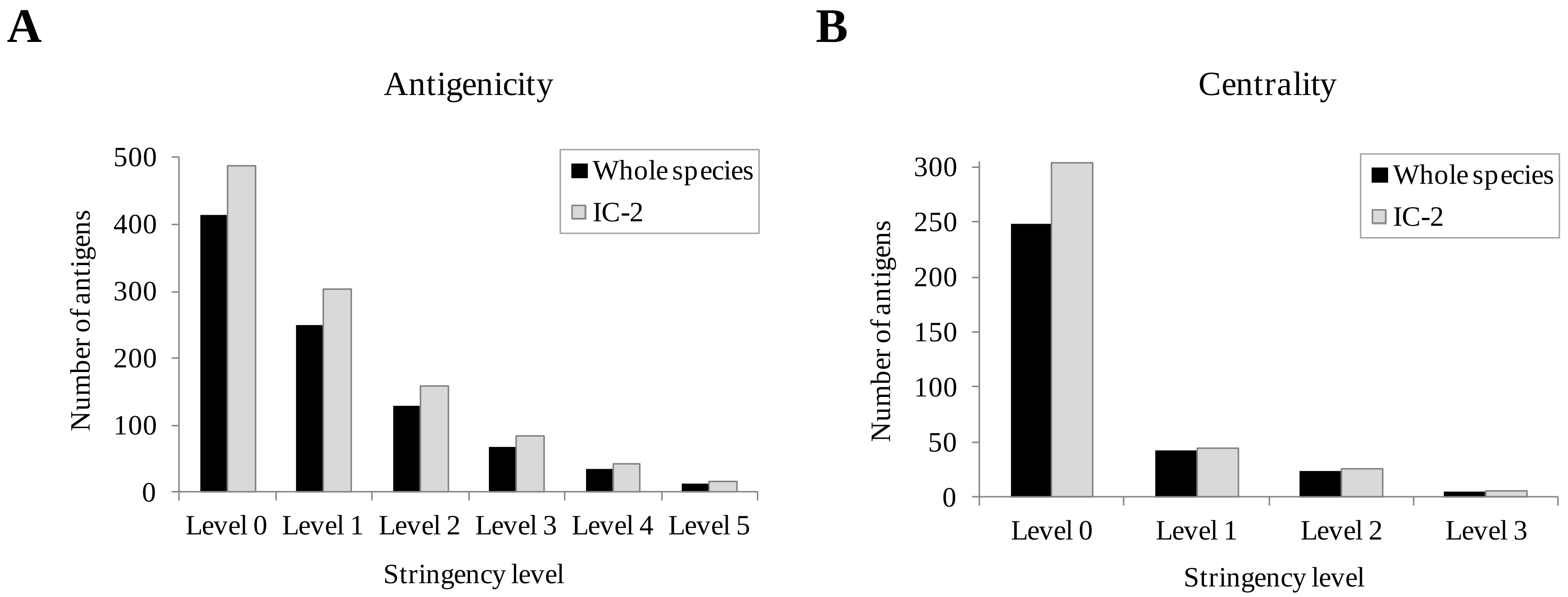

(A) Stringency levels:

Level	B-cell antigenic zones	DP supertype alleles (epitopes)
0	≥ 0	≥ 0 (0)
1	≥ 1	≥ 1 (2)
2	≥ 2	≥ 2 (4)
3	≥ 3	≥ 3 (6)
4	≥ 4	≥ 4 (8)
5	≥ 5	≥ 5 (10)

(B) Stringency levels:

Level	PPIs [a]	BC[b]
0	≥ 0	≥ 0
1	≥ 3	≥ 0.001
2	≥ 5	≥ 0.005
3	≥ 10	≥ 0.02

[a] PPIs: number of protein-protein interactions.

[b] BC: betweenness centrality.
