# Supplementary material for: Designing Multi-Antigen Vaccines Against Acinetobacter baumannii Using Systemic Approaches
Source: Front Immunol. 2021 Apr 16;12:666742. doi: 10.3389/fimmu.2021.666742 (PMC8085427; doi:10.3389/fimmu.2021.666742)
Supplement: Supplementary file 2 [file Image_2.pdf]

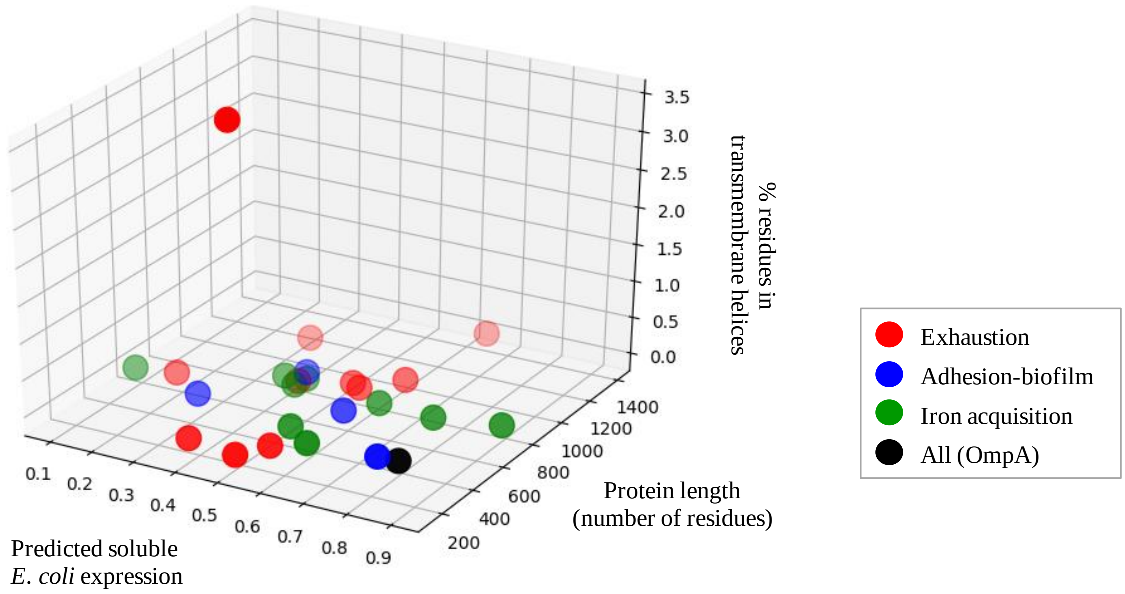

Figure S2. Predicted experimental behavior of selected immunotargets. The strategy followed for antigen selection is shown in the legend.
