# Supplementary material for: Designing Multi-Antigen Vaccines Against Acinetobacter baumannii Using Systemic Approaches
Source: Front Immunol. 2021 Apr 16;12:666742. doi: 10.3389/fimmu.2021.666742 (PMC8085427; doi:10.3389/fimmu.2021.666742)
Supplement: Supplementary file 3 [file Table_1.pdf]

Supplementary Table S1. Pfam domains utilized in this study.

Model	Pfam id	Description
BBP2	PF07642	Putative beta-barrel porin-2, OmpL-like. bbp2
CblD	PF07434	CblD like pilus biogenesis initiator
ChapFlgA	PF13144	Chaperone for flagella basal body P-ring formation
ComP_DUS	PF16732	Type IV minor pilin ComP, DNA uptake sequence receptor
CooC_C	PF15976	CS1-pili formation C-terminal
FlaE	PF07559	Flagellar basal body protein FlaE
Flagellin_C	PF00700	Bacterial flagellin C-terminal helical region
Flg_bb_rod	PF00460	Flagella basal body rod protein
Flg_bbr_C	PF06429	Flagellar basal body rod FlgEFG protein C-terminal
FlgD	PF03963	Flagellar hook capping protein - N-terminal region
FlgH	PF02107	Flagellar L-ring protein
FlgI	PF02119	Flagellar P-ring protein
FlhC	PF05280	Flagellar transcriptional activator (FlhC)
FlhD	PF05247	Flagellar transcriptional activator (FlhD)
FliE	PF02049	Flagellar hook-basal body complex protein FliE
FliH	PF02108	Flagellar assembly protein FliH
FliMN_C	PF01052	Type III flagellar switch regulator (C-ring) FliN C-term
FliS	PF02561	Flagellar protein FliS
Flp_Fap	PF04964	Flp/Fap pilin component
KdgM	PF06178	Oligogalacturonate-specific porin protein (KdgM)
LamB	PF02264	LamB porin
MscS_porin	PF12795	Mechanosensitive ion channel porin domain
Opacity	PF02462	Opacity family porin protein
OprB	PF04966	Carbohydrate-selective porin, OprB family
OprD	PF03573	Outer membrane porin, OprD family
PapD_C	PF02753	Pili assembly chaperone PapD, C-terminal domain
PapD_N	PF00345	Pili and flagellar-assembly chaperone, PapD N-terminal domain
PapD_N	PF00345	Pili and flagellar-assembly chaperone, PapD N-terminal domain
Pilin	PF00114	Pilin (bacterial filament)
PilJ	PF13675	Type IV pili methyl-accepting chemotaxis transducer N-term
PilM_2	PF11104	Type IV pilus assembly protein PilM
PilO	PF04350	Pilus assembly protein, PilO
PilP	PF04351	Pilus assembly protein, PilP
PilW	PF16074	Type IV Pilus-assembly protein W
Porin_1	PF00267	Gram-negative porin
Porin_2	PF02530	Porin subfamily
Porin_4	PF13609	Gram-negative porin
Porin_7	PF16956	Putative general bacterial porin
Porin_8	PF16966	Porin-like glycoporin RafY
Porin_O_P	PF07396	Phosphate-selective porin O and P
RcpC	PF16976	Flp pilus assembly protein RcpC/CpaB
Sugarporin_N	PF11471	Maltoporin periplasmic N-terminal extension
SWM_repeat	PF13753	Putative flagellar system-associated repeat

T2SSppdC	PF12528	Type II secretion prepilin peptidase dependent protein C
T2SS-T3SS_pil_N	PF13629	Pilus formation protein N terminal region
Tad	PF13400	Putative Flp pilus-assembly TadE/G-like
TraC_F_IV	PF11130	F pilus assembly Type-IV secretion system for plasmid transfer
TraQ	PF09679	Type-F conjugative transfer system pilin chaperone (TraQ)
TrbC_Ftype	PF09673	Type-F conjugative transfer system pilin assembly protein
YscJ_FliF_C	PF08345	Flagellar M-ring protein C-terminal
