# Supplementary material for: Designing Multi-Antigen Vaccines Against Acinetobacter baumannii Using Systemic Approaches
Source: Front Immunol. 2021 Apr 16;12:666742. doi: 10.3389/fimmu.2021.666742 (PMC8085427; doi:10.3389/fimmu.2021.666742)
Supplement: Supplementary file 4 [file Table_2.pdf]

Supplementary Table S2. Proteomic experiments using fractionated samples considered in this study.

Fraction	Number of identified proteins	Original strain	Reference
Cell envelope	23	A15-43	[1]
Extracellular	30	DU202	[2]
Outer membrane	74	DU202	[2]
Outer membrane vesicles	131	DU202	[3]
Outer membrane vesicles	179	AbH12O-A2	[4]
Periplasmic space	55	DU202	[2]
Secretome	146	AbH12O-A2	[4]

References

1. Martí *et al*. Proteomic analysis of a fraction enriched in cell envelope proteins of *Acinetobacter baumannii*. *Proteomics.* (2016) 6 Suppl 1: S82-7.

2. Yun *et al*. Quantitative proteomic analysis of cell wall and plasma membrane fractions from multidrug-resistant *Acinetobacter baumannii*. *J Proteome Res*. (2011) 10:459-69.

3. Kwon *et al*. Proteome analysis of outer membrane vesicles from a clinical *Acinetobacter baumannii* isolate. *FEMS Microbiol Lett*. (2009) 297:150-6.

4. Mendez *et al*. Extracellular proteome of a highly invasive multidrug-resistant clinical strain of *Acinetobacter baumannii*. *J Proteome Res*. (2012) 11:5678-94.
