# Supplementary material for: Designing Multi-Antigen Vaccines Against Acinetobacter baumannii Using Systemic Approaches
Source: Front Immunol. 2021 Apr 16;12:666742. doi: 10.3389/fimmu.2021.666742 (PMC8085427; doi:10.3389/fimmu.2021.666742)
Supplement: Supplementary file 5 [file Table_3.pdf]

Supplementary Table S3. Antigens statistically associated with different *A. baumannii* syndromes.

Syndrome	Protein	Description	% Occurrence in the syndrome	% Average occurrence in the rest of syndromes	*P* value
Bloodstream infection	LPP	Membrane protein	61.4	50.3	0.0444
	LprI	DUF1311 domain-containing protein (LprI lysozyme inhibitor)	67.5	55.7	0.0172
Respiratory tract infection	CirA	TonB-dependent siderophore receptor	71.2	62.3	0.0310
	RpsB	30S ribosomal protein S2	93.5	86.3	0.0139
	Smc	Hypothetical protein	94.2	84.9	0.0023
	WP_000608684.1	Hypothetical protein	61.9	47.9	0.0010
Wounds	DUF3261	DUF3261 domain-containing protein	60.0	40.2	0.0008
	MarB	Multiple antibiotic resistance protein MarB	74.3	60.0	0.0153
	PRK10053	YdeI family stress tolerance OB fold protein	75.7	63.5	0.0353
	TraH	Conjugal transfer protein TraH	58.6	41.1	0.0031
	WP_000827933.1	Hypothetical protein	72.9	55.3	0.0033
	WP_002358608.1	Hypothetical protein	72.9	60.7	0.0380
	WP_046661213.1	Hypothetical protein	74.3	60.0	0.0153
	WP_049067394.1	Hypothetical protein	54.3	37.6	0.0042
	YdeI	YgiW/YdeI family stress tolerance OB fold protein	61.4	40.7	0.0005
