# Supplementary material for: Designing Multi-Antigen Vaccines Against Acinetobacter baumannii Using Systemic Approaches
Source: Front Immunol. 2021 Apr 16;12:666742. doi: 10.3389/fimmu.2021.666742 (PMC8085427; doi:10.3389/fimmu.2021.666742)
Supplement: Supplementary file 6 [file Table_4.pdf]

Supplementary Table S4. Expression experiments considered in this study.

Condition	Experiment	Original strain	Number of upregulated or essential genes	Reference
Bacteremia	RNA-Seq	ATCC 17978	557	[1]
Bloodstream infection (required)	Tn-insertion	ATCC 17978	59	[2]
BALF	Proteomics	AbH12O-A2	50	[3]
Biofilm vs exponential planktonic cells	RNA-Seq	ATCC 17978	35	[4]
Biofilm vs stationary planktonic cells	RNA-Seq	ATCC 17978	116	[4]
Hydrogen peroxide	RNA-Seq	ATCC 17978	155	[5]
Iron limitation	Microarray	ATCC 17978	463	[6]
Macrophage infection	Proteomics	AbH12O-A2	76	[3]
Mucin 0.5%	RNA-Seq	ATCC 19606	232	[7]
Pneumonia (required)	Tn-insertion	ATCC 17978	157	[8]
Rich medium (required)	Tn-insertion	ATCC 17978	453	[8]
Stationary vs exponential planktonic cells	RNA-Seq	ATCC 17978	130	[4]

References

1. Murray *et al*. Global Gene Expression Profile of *Acinetobacter baumannii* During Bacteremia. *J Infect Dis*. (2017) 215:S52-S57.

2. Subashchandrabose *et al*. *Acinetobacter baumannii* Genes Required for Bacterial Survival during Bloodstream Infection. *mSphere*. (2015) 1: e00013-15.

3. Mendez *et al*. Quantitative proteomic analysis of host--pathogen interactions: a study of *Acinetobacter baumannii* responses to host airways. *BMC Genomics* (2015) 16:422.

4. Rumbo-Feal *et al*. Whole transcriptome analysis of *Acinetobacter baumannii* assessed by RNA-sequencing reveals different mRNA expression profiles in biofilm compared to planktonic cells. *PLoS One*. (2013) 8:e72968.

5. Juttukonda *et al*. *Acinetobacter baumannii* OxyR Regulates the Transcriptional Response to Hydrogen Peroxide. *Infect Immun*. (2018) 87:e00413-18.

6. Eijkelkamp *et al*. Investigation of the human pathogen *Acinetobacter baumannii* under iron limiting conditions. *BMC Genomics*. (2011) 12:126.

7. Ohneck *et al*. Mucin acts as a nutrient source and a signal for the differential expression of genes coding for cellular processes and virulence factors in *Acinetobacter baumannii*. *PLoS One*. (2018) 13: e0190599.

8. Wang *et al*. Genome-wide identification of *Acinetobacter baumannii* genes necessary for persistence in the lung. *mBio*. (2014) 5:e01163-14.
