# Supplementary material for: Designing Multi-Antigen Vaccines Against Acinetobacter baumannii Using Systemic Approaches
Source: Front Immunol. 2021 Apr 16;12:666742. doi: 10.3389/fimmu.2021.666742 (PMC8085427; doi:10.3389/fimmu.2021.666742)
Supplement: Supplementary file 7 [file Table_5.pdf]

Figure S5. Antigens satisfying high stringent selection.

Protein	Description	Occurrence (% isolates)	Number of B-cell epitopic zones	Number of DP supertype alleles (epitopes)	Number of PPIs[a]	Betweenness centrality
LptD	LPS-assembly protein LptD	99.42	4	5 (19)	5	0.038
OmpA	OmpA-like protein	96.87	5	2 (3)	28	0.208
WP_001043188.1	Hypothetical protein	99.66	1	3 (4)	32	0.027
PBP3	Penicillin-binding protein PBP3	99.42	5	5 (18)	7	0.029
LolA	Outer membrane lipoprotein chaperone LolA	99.59	1	5 (8)	5	0.035

[a] PPIs: number of protein-protein interactions.
