# Supplementary material for: Designing Multi-Antigen Vaccines Against Acinetobacter baumannii Using Systemic Approaches
Source: Front Immunol. 2021 Apr 16;12:666742. doi: 10.3389/fimmu.2021.666742 (PMC8085427; doi:10.3389/fimmu.2021.666742)
Supplement: Supplementary file 8 [file Table_6.pdf]

Table S6. List of antigens essential in rich medium.

Protein symbol	Occurrence (% isolates)	Number of B-cell epitopic zones	Number of DP supertype alleles (epitopes)	Number of PPIs	Betweenness centrality	Description
HemH	99.52	1	5 (13)	2	0	Ferrochelatase
DapD	99.44	1	3 (6)	1	0	2,3,4,5-tetrahydropyridine-2,6-carboxylate N-succinyltransferase
TonB	99.25	2	1 (2)	2	5×10^{-5}	Energy transducer TonB
RplF	99.78	1	1 (1)	37	0.002	50S ribosomal protein L6
HAD_like	99.85	1	5 (8)	2	0	Haloacid Dehalogenase-like Hydrolase
RpoA	99.66	2	3 (4)	35	0.004	DNA-directed RNA polymerase subunit alpha
RplU	99.83	1	1 (1)	35	0.002	50S ribosomal protein L21
RodZ	99.2	2	4 (5)	3	0	HTH domain-containing protein
SPOR	94.67	1	2 (2)	1	0	SPOR domain-containing protein
RpsC	99.78	1	2 (3)	39	0.007	30S ribosomal protein S3
Outer_YhbN_LptA	99.71	1	2 (2)	2	0	Lipopolysaccharide transport periplasmic protein LptA
LptD	99.42	4	5 (19)	5	0.039	LPS assembly outer membrane protein LptD
WP_000816400.1	92.66	1	3 (5)	1	0	Putative 2-aminoethylphosphonate ABC transporter substrate-binding protein
CTP_transf_1	99.69	1	5 (27)	1	0	Phosphatidate cytidylyltransferase
MurD	99.49	2	5 (9)	3	0.011	UDP-N-acetylmuramoyl-L-alanine--D-glutamate ligase
CtpA	99.39	2	5 (13)	0	0	Peptidase S41

RibH	99.81	2	5 (9)	1	0	6,7-dimethyl-8-ribityllumazine synthase
SecD	98.55	1	5 (19)	0	0	SecD export protein N-terminal TM region
GlnA	99.47	2	5 (13)	4	0.001	Type I glutamate--ammonia ligase
RpoC	98.89	3	5 (33)	29	0.003	DNA-directed RNA polymerase subunit beta
LptC	99.73	2	1 (1)	32	0.048	LPS export ABC transporter periplasmic protein LptC
PpsA	99.25	2	5 (27)	8	0.013	Phosphoenolpyruvate synthase
Pbp3	99.42	5	5 (18)	7	0.029	Penicillin-binding protein PBP3
LolA	99.59	1	5 (8)	5	0.035	Outer membrane lipoprotein chaperone LolA
