# Supplementary material for: Designing Multi-Antigen Vaccines Against Acinetobacter baumannii Using Systemic Approaches
Source: Front Immunol. 2021 Apr 16;12:666742. doi: 10.3389/fimmu.2021.666742 (PMC8085427; doi:10.3389/fimmu.2021.666742)
Supplement: Supplementary file 9 [file Table_7.pdf]

Table S7. Predicted experimental properties of selected immunotargets.

Selection strategy	Targeted mechanism	Protein	Protein length	Soluprot score	% residues in transmembane helices
All	All	WP_000777882.1	356	0.806	0
Siege	Adhesion-biofilm	WP_001061322.1	609	0.586	0
Siege	Adhesion-biofilm	WP_004644147.1	352	0.759	0
Siege	Adhesion-biofilm	WP_017386534.1	491	0.281	0
Siege	Adhesion-biofilm	WP_096903805.1	819	0.420	0
Siege	Iron acquisition	WP_000364460.1	710	0.431	0
Siege	Iron acquisition	WP_000413985.1	772	0.437	0
Siege	Iron acquisition	WP_000831228.1	704	0.763	0
Siege	Iron acquisition	WP_000848134.1	417	0.532	0
Siege	Iron acquisition	WP_000871878.1	571	0.096	0
Siege	Iron acquisition	WP_001189913.1	718	0.631	0
Siege	Iron acquisition	WP_050675416.1	330	0.603	0
Siege	Iron acquisition	WP_079746199.1	760	0.903	0
Siege	Iron acquisition	WP_115431403.1	759	0.390	0
Exhaustion	Exhaustion	WP_000632986.1	742	0.428	0
Exhaustion	Exhaustion	WP_000682636.1	817	0.532	0
Exhaustion	Exhaustion	WP_000701694.1	176	0.383	0
Exhaustion	Exhaustion	WP_000733830.1	604	0.185	0
Exhaustion	Exhaustion	WP_000809155.1	672	0.293	3.42
Exhaustion	Exhaustion	WP_001043188.1	140	0.507	0
Exhaustion	Exhaustion	WP_001218018.1	255	0.545	0
Exhaustion	Exhaustion	WP_004781676.1	1399	0.648	0
Exhaustion	Exhaustion	WP_017392669.1	1074	0.333	0
Exhaustion	Exhaustion	WP_020753375.1	924	0.619	0
Exhaustion	Exhaustion	WP_044697869.1	794	0.556	0
