# Supplementary material for: Designing Multi-Antigen Vaccines Against Acinetobacter baumannii Using Systemic Approaches
Source: Front Immunol. 2021 Apr 16;12:666742. doi: 10.3389/fimmu.2021.666742 (PMC8085427; doi:10.3389/fimmu.2021.666742)
Supplement: Supplementary file 10 [file Table_8.pdf]

Table S8. Predicted toxicity and allergenicity of selected immunotargets.

Selection strategy	Targeted mechanism	Protein	% residues in toxic regions	Allergenicity					
					AlgPred 2.0				
				AllerTop 2.0	Mapping IgE epitopes	MAST result	SVM single	SVM dipeptide	Blast ARPS
All	All	WP_000777882.1	0.00	Probable non-allergen	No	No	YES	YES	No
Siege	Adhesion-biofilm	WP_004644147.1	0.00	Probable allergen	No	No	No	No	No
Siege	Adhesion-biofilm	WP_096903805.1	1.22	Probable non-allergen	No	No	YES	YES	No
Siege	Adhesion-biofilm	WP_001061322.1	0.00	Probable non-allergen	No	No	YES	YES	No
Siege	Adhesion-biofilm	WP_017386534.1	0.00	Probable allergen	No	No	No	No	No
Siege	Iron acquisition	WP_000871878.1	6.30	Probable non-allergen	No	No	No	No	No
Siege	Iron acquisition	WP_050675416.1	3.63	Probable non-allergen	No	No	Potential	YES	No
Siege	Iron acquisition	WP_000848134.1	0.00	Probable non-allergen	No	No	YES	Potential	No
Siege	Iron acquisition	WP_000413985.1	2.20	Probable non-allergen	No	No	Potential	Potential	No
Siege	Iron acquisition	WP_115431403.1	1.58	Probable non-allergen	No	No	Potential	Potential	No
Siege	Iron acquisition	WP_000364460.1	3.38	Probable allergen	No	No	Potential	YES	No
Siege	Iron acquisition	WP_000831228.1	0.00	Probable non-allergen	No	No	Potential	Potential	No
Siege	Iron acquisition	WP_001189913.1	0.00	Probable non-allergen	No	No	Potential	Potential	No
Siege	Iron acquisition	WP_079746199.1	0.00	Probable non-allergen	No	No	YES	Potential	No
Exhaustion	Exhaustion	WP_000632986.1	7.14	Probable allergen	No	No	YES	YES	No
Exhaustion	Exhaustion	WP_000682636.1	6.36	Probable non-allergen	No	No	Potential	Potential	No
Exhaustion	Exhaustion	WP_000701694.1	0.00	Probable non-allergen	No	No	YES	YES	No
Exhaustion	Exhaustion	WP_000733830.1	4.14	Probable non-allergen	No	No	No	No	No
Exhaustion	Exhaustion	WP_000809155.1	0.00	Probable non-allergen	No	No	No	YES	No
Exhaustion	Exhaustion	WP_001043188.1	0.00	Probable non-allergen	No	No	YES	No	No
Exhaustion	Exhaustion	WP_001218018.1	0.00	Probable non-allergen	No	No	Potential	YES	No
Exhaustion	Exhaustion	WP_004781676.1	4.29	Probable allergen	No	No	No	No	No
Exhaustion	Exhaustion	WP_017392669.1	0.00	Probable non-allergen	No	No	No	No	No

Exhaustion	Exhaustion	WP_020753375.1	1.08	Probable non-allergen	No	No	YES	Potential	No
Exhaustion	Exhaustion	WP_044697869.1	1.39	Probable non-allergen	No	No	YES	No	No
